# Age at Diagnosis of Atrial Fibrillation and Incident Dementia

**DOI:** 10.1001/jamanetworkopen.2023.42744

**Published:** 2023-11-08

**Authors:** Wenya Zhang, Jie Liang, Chenglong Li, Darui Gao, Qian Ma, Yang Pan, Yongqian Wang, Wuxiang Xie, Fanfan Zheng

**Affiliations:** 1 Department of Clinical Nursing, School of Nursing, Chinese Academy of Medical Sciences & Peking Union Medical College, Beijing, China; 2 Heart and Vascular Health Research Center, Peking University Clinical Research Institute, Peking University First Hospital, Beijing, China; 3 Key Laboratory of Epidemiology of Major Diseases (Peking University), Ministry of Education, Beijing, China; 4 Department of Cardiology, Beijing Anzhen Hospital, Capital Medical University, Beijing, China

## Abstract

**Importance:**

Epidemiological evidence regarding the association between atrial fibrillation (AF) onset age and risk of incident dementia remains unexplored.

**Objective:**

To examine whether age at AF diagnosis is associated with risk of incident dementia and its subtypes.

**Design, Setting, and Participants:**

This prospective, population-based cohort study used data from UK Biobank, a public, open-access database in the UK with baseline information collected from 2006 to 2010. A total of 433 746 participants were included in the main analysis after excluding participants with a diagnosis of dementia or AF at baseline, missing data on covariates, or having dementia before AF onset during a median follow-up of 12.6 years. Data were analyzed from October to December 2022.

**Exposures:**

AF diagnosis and age at AF diagnosis according to age groups (<65 years, 65-74 years, or ≥75 years).

**Main Outcomes and Measures:**

Incident dementia, ascertained through linkage from multiple databases until December 31, 2021. Cox proportional hazards models and the propensity score matching method were adopted to estimate the association between AF onset age and incident dementia.

**Results:**

Of 433 746 included participants, 236 253 (54.5%) were female, the mean (SD) age was 56.9 (8.1) years, and 409 990 (94.5%) were White. Compared with individuals without AF, 30 601 individuals with AF had a higher risk of developing all-cause dementia (adjusted hazard ratio [HR], 1.42; 95% CI, 1.32-1.52) and vascular dementia (VD; adjusted HR, 2.06; 95% CI, 1.80-2.36), but not Alzheimer disease (AD; adjusted HR, 1.08; 95% CI, 0.96-1.21). Among participants with AF, younger age at AF onset was associated with higher risks of developing all-cause dementia (adjusted HR per 10-year decrease, 1.23; 95% CI, 1.16-1.32), AD (adjusted HR per 10-year decrease, 1.27; 95% CI, 1.13-1.42), and VD (adjusted HR per 10-year decrease, 1.35; 95% CI, 1.20-1.51). After propensity score matching, individuals with AF diagnosed before age 65 years had the highest HR of developing all-cause dementia (adjusted HR, 1.82; 95% CI, 1.54-2.15), followed by AF diagnosed at age 65 to 74 years (adjusted HR, 1.47; 95% CI, 1.31-1.65) and diagnosed at age 75 years or older (adjusted HR, 1.11; 95% CI, 0.96-1.28). Similar results can be seen in AD and VD.

**Conclusions and Relevance:**

In this prospective cohort study, earlier onset of AF was associated with an elevated risk of subsequent all-cause dementia, AD, and VD, highlighting the importance of monitoring cognitive function among patients with AF, especially those younger than 65 years at diagnosis.

## Key Points

**Question:**

Is there an association between atrial fibrillation (AF) onset age and incident dementia?

**Findings:**

In this prospective cohort study including 433 746 UK Biobank participants, younger age at AF diagnosis was associated with incident all-cause dementia, Alzheimer disease, and vascular dementia after multivariable adjustment, which was additionally strengthened by the results of propensity score matching.

**Meaning:**

The findings indicate that careful monitoring of cognitive function for patients with a younger AF onset age, particularly those diagnosed with AF before age 65 years, is important to attenuate the risk of subsequent dementia.

## Teaser

This cohort study examines whether age at diagnosis of atrial fibrillation is associated with risk of incident dementia and its subtypes.

## Introduction

Dementia remains a major and increasing health challenge globally, considering its high mortality and huge public health burdens.^1^ Moreover, the prevalence and incidence of dementia tend to steadily increase with the longer life expectancy of the population and the accumulation of predisposing factors.^2^ Nevertheless, there is no effective pharmacological intervention that prevents or reverses the pathological damage and degeneration of neurons in dementia.^3^ Overall, a growing body of evidence supports that effective interventions for risk factors might contribute to delaying dementia onset and reducing the number of people developing dementia in the future.^4,5,6^

Atrial fibrillation (AF) is the most common sustained cardiac arrhythmia worldwide, which often coexists and might be related to dementia in various aspects.^7,8^ First, complications from AF tend to cause stroke, which is proven to be an essential risk factor for dementia.^6,9^ Second, there are several proposed mechanisms linking AF and subsequent cognitive decline, including AF-related cerebral infarction, hypoperfusion, inflammation, atherosclerotic vascular disease, microhemorrhage, and brain atrophy.^10,11^ Third, AF shares risk factors with dementia, including advanced age, obesity, diabetes, excessive alcohol use, and others.^9,12^

Accumulating epidemiological findings indicated that the risks of subsequent cognitive impairment and dementia appeared to be higher in people living with AF,^13,14,15,16,17,18,19^ but no clear consensus has been achieved on whether having AF diagnosed earlier in the life course is associated with increased risk of developing dementia in later life. A recent report from the Coronary Artery Risk Development in Young Adults (CARDIA) study ascertained that premature cardiovascular disease (defined as ≤60 years) was associated with worse midlife cognition and white matter health, irrespective of stroke, transient ischemic attack, and cardiovascular risk factors.^20^ Moreover, longer exposure to AF appears to increase the risk of incident dementia.^15,16,19^ However, these studies either focused on the influence of cardiovascular health on midlife cognitive function or the association between exposure duration after AF diagnosis and incident dementia, so the association between AF diagnosed at different stages across the lifespan and incident dementia in later life remains unexplored. Based on previous evidence, it is plausible for us to assume that people with younger onset age of AF, a common type of cardiac arrhythmia, have a higher risk of developing dementia.

Identifying the association between AF onset age and incident dementia may provide support for early monitoring of cognitive function in patients with AF, which in turn delays or prevents the development of dementia in later life. Hence, by using data from the UK Biobank (UKB), in which data on AF diagnosis, incident dementia, and relevant covariates were collected for a large and prospective cohort, we aimed to explore whether younger onset age of AF was associated with a higher risk of incident dementia.

## Methods

### Study Design and Population

Our study used data from the UKB, a public, open-access database in the UK that explores the associations between genetic factors, environmental factors, lifestyle habits, and major human diseases. UKB investigators initially sent more than 9 million postal invitations to residents aged 40 to 69 years who registered with the UK’s National Health Service and lived within 25 miles of 1 of 22 assessment centers located throughout England, Wales, and Scotland. Ultimately, 502 411 participants consented to join the prospective cohort study and visited an assessment center between 2006 to 2010 for baseline assessments, resulting in a participation rate of 5.4%.^21^ Data collection process included a touchscreen questionnaire, verbal interview, physical measures, and biosample collection, allowing researchers to gain an in-depth knowledge of sociodemographic, physical, lifestyle, and health-related anonymized data about UKB participants. Other detailed information on data collection can be found on the UKB website^22^ and published articles.^21,23,24^ Ethical consent was approved by the NHS North West Multicenter Research Ethical Committee, and all participants provided informed consent before baseline data collection. The report of our study followed the Strengthening the Reporting of Observational Studies in Epidemiology (STROBE) reporting guideline.

The Figure shows the flowchart of participant selection of this study. In short, among 502 411 participants recruited at baseline, we excluded participants with baseline dementia or stroke (n = 8689), missing data on covariates (n = 58 905), or having dementia before AF onset during follow-up visits (n = 1071). The remaining 433 746 participants were included in the analyses to explore whether AF was associated with an increased risk of developing dementia. Next, 30 601 participants with data on age of AF onset were included in the analyses to investigate the association between onset age of AF and incident dementia. Finally, 30 600 participants with AF and 61 200 matched participants without AF (1:2 matching) were included in propensity score matching analyses to investigate the association between AF and incident dementia among different AF onset age groups.

**Figure.**
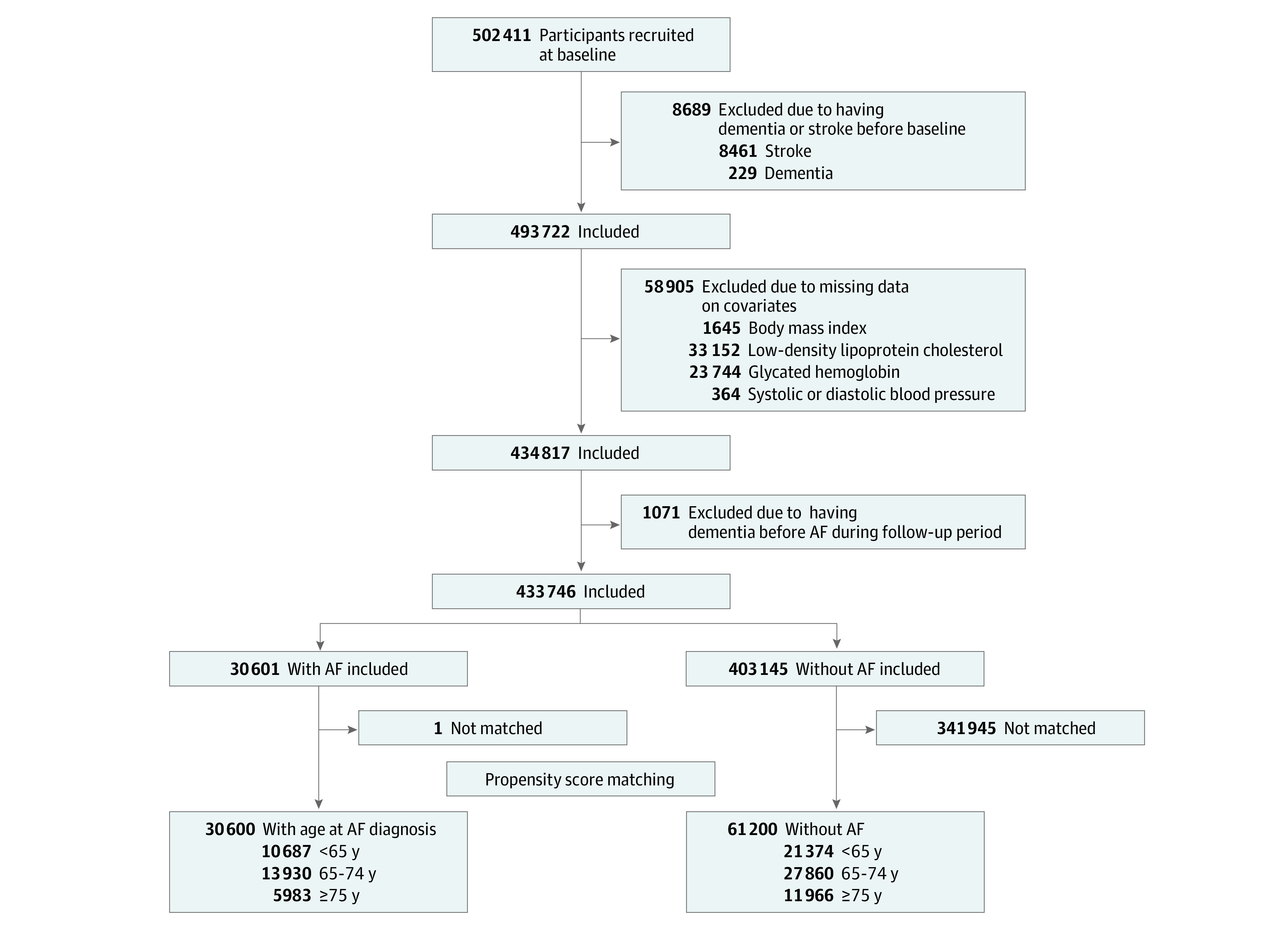
Flowchart of Participant Selection AF indicates atrial fibrillation.

### Ascertainment of AF and Age at AF Diagnosis

AF was identified using first occurrence data (Field identification [ID]: 131350) with the *International Statistical Classification of Diseases and Related Health Problems, Tenth Revision *(*ICD-10*) codes of I48, which were derived from baseline self-reported data, primary care data, hospital inpatient data, and death registry records.^25^ The age at diagnosis of AF was calculated as subtracting birth date from the initial date of AF diagnosis and dividing by 365.

### Ascertainment of Dementia

Cases of all-cause dementia, Alzheimer disease (AD), and vascular dementia (VD) were ascertained using algorithmically defined outcomes through linkage from baseline self-reported data, hospital admissions data, and death registry records (Field IDs: 42018, 42020, 42022, and 42024).^26^ eTable 1 in Supplement 1 presents *ICD-10* codes used to define dementia, and the earliest recorded date regardless of sources was considered the onset date of dementia.

### Covariates

Covariates included age, sex, ethnicity (White or other ethnicity [defined by UKB as Asian, Black, Chinese, mixed, or other ethnicity and combined because of small sample size]), educational level (higher educational level or not), current smoking (yes or no), current drinking (at least once per week or not), physical activity (yes or no), and depressed mood (yes or no). Higher educational level refers to a college or university degree or other professional qualifications. Physical activity was defined as moderate or vigorous physical activity for more than 10 minutes with a frequency of at least twice per week. Depressed mood was defined if a participant reported a sense of depression or hopelessness nearly every day or more than half the days over the past 2 weeks. Body mass index (BMI) was calculated as weight in kilograms divided by height in meters squared. Low-density lipoprotein cholesterol (LDL-C) was measured at baseline via direct enzymatic methods.^27^ Use of antihypertensive drugs (yes or no), antihyperglycemic drugs (yes or no), and statins (yes or no) was obtained at baseline touchscreen questionnaire or verbal interview. Hypertension was defined according to baseline self-reported hypertension, use of antihypertensive medication, or baseline physical measures (mean systolic blood pressure [SBP]/diastolic blood pressure [DBP] ≥140/90 mm Hg). Diabetes was defined based on self-reported diabetes (diabetes, type 1 diabetes or type 2 diabetes), use of antihyperglycemic medication or baseline physical measures (plasma glycated hemoglobin [HbA_1c_] ≥48 mmol/mol or ≥6.5% [to convert to proportion of total hemoglobin, multiply by 0.01]). Coronary heart disease (CHD) was defined according to baseline self-reported data and verbal interviews with trained staff. apolipoprotein E4 (APOE4) status (carrier, noncarrier) was based on genomic data.^28^ Additional detailed information about the covariates is summarized in eTable 2 in Supplement 1.

### Statistical Analysis

The baseline characteristics of 433 746 participants were presented as the mean with SD for continuous variables and frequency and percentage for categorical variables. Comparison of baseline characteristics between the AF group (participants with a diagnosis of AF before baseline or during follow-up) and the non-AF group was tested via a *t* test or χ^2^ test. The associations of AF and its onset age with incident dementia were investigated using multivariate Cox proportional hazard models with years between baseline to incident dementia, death, or follow-up, whichever came first, as the time scale. All results were displayed as hazard ratios (HRs) with 95% CIs. First, we tested the association of AF with incident dementia among the total population (N = 433 746). Next, we divided participants with an onset age of AF (n = 30 601) into 3 age groups (<65, 65-74, ≥75 years) and tested the associations between AF onset age and incident all-cause dementia, AD, and VD, with the group aged 75 years or older as the reference. Finally, propensity score matching with the nearest-neighbor method was performed within each age group so that every participant in these 3 age groups was matched with 2 participants without AF who were randomly selected from the AF-free population (n = 403 145). The associations of AF with incident all-cause dementia, AD, and VD were examined within each age group. The propensity score matching analyses were adjusted for the following variables: age, sex, ethnicity, education, BMI, LDL-C, SBP, DBP, smoking, alcohol use, physical activity, depressed mood, hypertension, diabetes, CHD, statin use, and APOE4 status.

Moreover, we conducted several sensitivity analyses. First, to avoid overestimation of the magnitude of association, AF was treated as a time-varying variable in the Cox proportional hazard model to assess the robustness of the results from main analyses. Second, after handling missing covariate data via multiple imputation, the association of AF onset age and incident all-cause dementia and its subtypes was explored among 34 822 participants with AF and complete covariates data. Third, the competing risk model was adopted to examine how treating death as a competing event affected the association between AF and incident dementia, which is an appropriate method to estimate competing actual risks.^29^ Fourth, to avoid possible reverse causality, we excluded incident dementia cases within the first 5 years of follow-up.^30^ Fifth, because of the relatively low prevalence of dementia among the younger population, we excluded participants with a baseline age younger than 50 years and performed the same main analyses. Sixth, given the possible disruption or delay of diagnoses of chronic diseases during the COVID-19 pandemic, we excluded incident dementia cases confirmed later than December 31, 2019, to alleviate the impact of this pandemic on our results. Finally, to estimate the robustness of these associations, subgroup analyses were performed to distinguish potential modifying factors.

We performed all statistical analyses via SAS version 9.4 (SAS Institute). Statistical significance was set at *P* < .05. Data were analyzed from October to December 2022.

## Results

### Baseline Characteristics

Table 1 presents the characteristics of 433 746 participants (mean [SD] age, 56.9 [8.1] years; 236 253 [54.5%] female; 409 990 [94.5%] White) included in the final analyses. A total of 30 601 participants had AF at baseline or developed AF during the follow-up period. Compared with participants without AF, participants with AF were older (mean [SD] age, 56.5 [8.1] years vs 62.3 [6.1] years) and had a higher proportion of men (178 083 [44.2%] vs 19 410 [63.4%]), White patients (380 294 [94.3%] vs 29 696 [97.0%]), low education (211 333 [52.4%] vs 17 910 [58.5%]), hypertension (214 815 [53.3%] vs 22 462 [73.4%]), diabetes (21 919 [5.4%] vs 3543 [11.6%]), CHD (14 567 [3.6%] vs 4245 [13.9%]), use of antihypertensive drugs (74 067 [18.4%] vs 12 826 [41.9%]), use of antihyperglycemic drugs (12 747 [3.2%] vs 2139 [7.0%]), and use of statins (54 046 [13.4%] vs 9360 [30.6%]).

**Table 1.** Baseline Characteristics of the Study Participants, by History of AF at Baseline or During Follow-Up

Characteristic	Participants, No. (%) (N = 433 746)	
	AF (n = 30 601)	Non-AF (n = 403 145)
Age, mean (SD), y	62.3 (6.1)	56.5 (8.1)
Sex		
Female	11 191 (36.6)	225 062 (55.8)
Male	19 410 (63.4)	178 083 (44.2)
Ethnicity		
White^a^	29 696 (97.0)	380 294 (94.3)
Other^b^	905 (3.0)	22 851 (5.7)
Higher education	12 691 (41.5)	191 812 (47.6)
Current smoking	3027 (9.9)	42 080 (10.4)
Current drinking	21 543 (70.4)	280 106 (69.5)
Exercise	23 570 (77.0)	315 636 (78.3)
BMI, mean (SD)	28.9 (5.3)	27.3 (4.7)
SBP, mean (SD), mm Hg	142.6 (19.4)	137.5 (18.5)
DBP, mean (SD), mm Hg	82.8 (10.6)	82.2 (10.1)
HbA_1c_, mean (SD), mmol/mol	37.77 (8.1)	35.92 (6.5)
LDL-C, mean (SD), mg/dL	129.34 (34.75)	138.61 (34.75)
Depressed mood	1356 (4.4)	18 928 (4.7)
Hypertension	22 462 (73.4)	214 815 (53.3)
Diabetes	3543 (11.6)	21 919 (5.4)
Coronary heart disease	4245 (13.9)	14 567 (3.6)
Use of antihypertensive drugs	12 826 (41.9)	74 067 (18.4)
Use of antihyperglycemic drugs	2139 (7.0)	12 747 (3.2)
Use of statin	9360 (30.6)	54 046 (13.4)
APOE4 carrier	7162 (23.4)	96 700 (23.4)

Abbreviations: AF, atrial fibrillation; APOE4, apolipoprotein E4; BMI, body mass index (calculated as weight in kilograms divided by height in meters squared); DBP, diastolic blood pressure; HbA_1c_, glycated hemoglobin; LDL-C, low-density lipoprotein cholesterol; SBP, systolic blood pressure.

SI conversion: To convert LDL-C to millimoles per liter, multiply by 0.0259.

^a^ White included British, Irish, and any other White background.

^b^ Other was defined as Asian, Black, Chinese, mixed, or other ethnic background.

### AF Status and Incident Dementia

Over a median (IQR) follow-up of 12.6 (12.1-13.6) years, new-onset dementia occurred in 5898 participants (1.36% of the whole population), including 2546 AD and 1211 VD. Specifically, 1031 dementia cases (3.37%) were observed among 30 601 participants with AF, including 350 AD and 320 VD. Table 2 presents the outcomes of multivariate Cox proportional hazard models. Participants with AF presented significantly higher risks of developing all-cause dementia (adjusted HR, 1.42; 95% CI, 1.32-1.52, *P* < .001) and VD (adjusted HR, 2.06; 95% CI, 1.80-2.36, *P* < .001). However, there was no additional risk of developing AD among participants with AF (adjusted HR, 1.08; 95% CI, 0.96-1.21, *P* = .20).

**Table 2.** Associations of AF With Incident All-Cause Dementia, Alzheimer Disease, and Vascular Dementia Among All 433 746 Total Participants

Outcome	Patients with vs without AF, HR (95% CI)	*P* value
**All-cause dementia**		
Model 1^a^	1.51 (1.41-1.62)	<.001
Model 2^b^	1.42 (1.32-1.52)	<.001
**Alzheimer disease**		
Model 1^a^	1.11 (0.99-1.25)	.07
Model 2^b^	1.08 (0.96-1.21)	.20
**Vascular dementia**		
Model 1^a^	2.37 (2.08-2.71)	<.001
Model 2^b^	2.06 (1.80-2.36)	<.001

Abbreviations: AF, atrial fibrillation; HR, hazard ratio.

^a^ Adjusted for age, sex, ethnicity, and education.

^b^ Further adjusted for baseline body mass index, low-density lipoprotein cholesterol level, current smoking, current drinking, exercise, depressed mood, hypertension, diabetes, coronary heart disease, use of antihypertensive drugs, use of antihyperglycemic drugs, use of statin, and apolipoprotein E4 status.

### Age at Diagnosis of AF and Incident Dementia

As shown in Table 3, the onset age of AF was significantly associated with an increased risk of developing dementia among participants with AF, which indicated that participants with younger age at diagnosis of AF had higher risks of developing all-cause dementia (adjusted HR per 10-year decrease, 1.23; 95% CI, 1.16-1.32, *P* < .001), AD (adjusted HR per 10-year decrease, 1.27; 95% CI, 1.13-1.42, *P* < .001), and VD (adjusted HR per 10-year decrease, 1.35; 95% CI, 1.20-1.51, *P* < .001).

**Table 3.** Associations of AF With Incident All-Cause Dementia, Alzheimer Disease, and Vascular Dementia Among 30 601 Participants With AF, by Onset Age Group

Outcome	HR (95% CI)^a^	*P* value
**All-cause dementia**		
≥75 y (n = 5984)	1 [Reference]	NA
65-74 y (n = 13 930)	1.46 (1.25-1.69)	<.001
<65 y (n = 10 687)	1.74 (1.45-2.09)	<.001
Per 10-y decrease	1.23 (1.16-1.32)	<.001
**Alzheimer disease**		
≥75 y (n = 5984)	1 [Reference]	NA
65-74 y (n = 13 930)	1.44 (1.12-1.86)	<.001
<65 y (n = 10 687)	1.76 (1.29-2.39)	.005
Per 10-y decrease	1.27 (1.13-1.42)	<.001
**Vascular dementia**		
≥75 y (n = 5984)	1 [Reference]	NA
65-74 y (n = 13 930)	2.05 (1.55-2.73)	<.001
<65 y (n = 10 687)	2.18 (1.54-3.08)	<.001
Per 10-y decrease	1.35 (1.20-1.51)	<.001

Abbreviations: AF, atrial fibrillation; HR, hazard ratio; NA, not applicable.

^a^ Adjusted for age, sex, ethnicity, education, baseline body mass index, low-density lipoprotein cholesterol level, current smoking, current drinking, exercise, depressed mood, hypertension, diabetes, coronary heart disease, use of antihypertensive drugs, use of antihyperglycemic drugs, use of statin, and apolipoprotein E4 status.

### AF and Incident Dementia After Propensity Score Matching

As shown in eTable 3 in Supplement 1, after propensity score matching, baseline characteristics of participants with or without AF were similar in most covariates. The risks of all-cause dementia, AD, and VD by age at diagnosis of AF are displayed in Table 4. Compared with individuals without AF, individuals with AF diagnosed at younger than 65 years had the highest HR for developing all-cause dementia (adjusted HR, 1.82; 95% CI, 1.54-2.15, *P* < .001), followed by AF diagnosed at age 65 to 74 years (adjusted HR, 1.47; 95% CI, 1.31-1.65, *P* < .001) and diagnosed at age 75 years or older (adjusted HR, 1.11; 95% CI, 0.96-1.28, *P* = .18). Similar results can be seen in AD and VD.

**Table 4.** Associations of AF With Incident All-Cause Dementia, Alzheimer Disease, and Vascular Dementia Among 91 800 Participants After Propensity Score Matching, by Onset Age Group

Outcome	Participants with vs without AF, HR (95% CI)^a^	*P* value
**All-cause dementia**		
≥75 y (n = 17 949)	1.11 (0.96-1.28)	.18
65-74 y (n = 41 790)	1.47 (1.31-1.65)	<.001
<65 y (n = 32 061)	1.82 (1.54-2.15)	<.001
**Alzheimer disease**		
≥75 y (n = 17 949)	0.83 (0.66-1.04)	.11
65-74 y (n = 41 790)	1.10 (0.91-1.33)	.30
<65 y (n = 32 061)	1.38 (1.05-1.82)	.02
**Vascular dementia**		
≥75 y (n = 17 949)	1.24 (0.92-1.68)	.16
65-74 y (n = 41 790)	2.31 (1.85-2.86)	<.001
<65 y (n = 32 061)	3.10 (2.21-4.36)	<.001

Abbreviations: AF, atrial fibrillation; HR, hazard ratio.

^a^ Adjusted for age, sex, ethnicity, education, baseline body mass index, low-density lipoprotein cholesterol level, current smoking, current drinking, exercise, depressed mood, hypertension, diabetes, coronary heart disease, use of antihypertensive drugs, use of antihyperglycemic drugs, use of statin, and apolipoprotein E4 status.

### Sensitivity Analysis

In general, the results of the sensitivity analysis remained stable compared with the results of the main analyses (eTables 4-15 in Supplement 1). Moreover, according to eFigures 1 to 5 in Supplement 1, subgroup analyses identified that ethnicity and APOE4 carrier status might modify the association between AF and incident dementia.

## Discussion

Our research showed that AF participants had an elevated risk of subsequent dementia compared with participants without AF. More importantly, multivariate Cox regression models indicated that an earlier diagnosis of AF was associated with greater risks of incident all-cause dementia, AD, and VD. Additionally, the results remained robust after propensity score matching, reinforcing the fact that the probability of developing dementia increases with a younger onset age of AF.

Ott et al^13^ proposed a noted, cross-sectional association of AF with dementia in 1997, and since then numerous studies have been conducted to investigate this association from epidemiological findings to pathological mechanisms while yielding inconsistent results.^14,15,16,17,18,19,31,32^ A meta-analysis^8^ published in 2021 demonstrated that patients with AF had a 60% higher risk of incident all-cause dementia, a 40% higher risk of incident AD, and a 70% higher risk of incident VD. These estimated HRs were partially aligned with our findings, which showed an HR of 1.38 for all-cause dementia and an HR of 1.95 for VD based on a large cohort with more than 30 000 participants with AF, suggesting that AF probably induced a more dramatic influence on the neuropathological cascades of VD.

To our knowledge, while current epidemiological studies still focus predominantly on the association between AF and subsequent cognitive decline or incident dementia, the present study is the largest study to explore the association between AF onset age and incident dementia. Based on accurate data on AF diagnoses and incident dementia, the most distinguished finding of our research was that an earlier diagnosis of AF was associated with an elevated risk of developing all-cause dementia, AD, and VD, which paralleled the findings from several prior studies exploring the risk of dementia or cognitive decline in AF patients.^15,16,19^ The Whitehall II study revealed an accelerated cognitive decline in those with longer exposure to AF (15 years), and this acceleration cannot be explained by incident stroke or coronary heart disease.^16^ By dividing participants into different groups according to their age at baseline, the Rotterdam study discovered a strong association between duration of exposure to AF (≤6 years, 6-12 years, and >12 years) and incident dementia as well as AD that was independent of stroke among the younger population (baseline age <67 years), while this association became insignificant among the older population (baseline age ≥67 years).^15^ Moreover, the results from the Intermountain Heart Collaborative Study suggested that people in the younger age group (baseline age <70 years) had the highest relative risk of incident all-cause dementia, senile dementia, AD, and VD.^19^ These findings collectively provide epidemiological evidence to reinforce the suspected mechanism that the earlier presence of AF may trigger progressive neuronal damage through repeated cerebral microembolisms and subclinical microinfarcts, causing longer exposure to cerebral hypoperfusion, hence resulting in chronic hypoxemia and ischemia-induced molecular events that would eventually lead to dementia.^33^ In addition, since AF and dementia share numerous risk factors, a younger onset age of AF means an earlier initiation of accumulating risk factors, which collectively exacerbate cognitive impairment.^11^

The primary strength of our study was the large sample size, enabling sufficient statistical power to investigate the association of AF onset age with incident dementia and its subtypes. Moreover, algorithmically defined health outcomes irrespective of source in UKB were revised and confirmed via a standardized approach, which ensured the precise ascertainment of the earliest diagnoses of AF and dementia, leading to a high positive predictive value of dementia cases (82.5%).^34^ Finally, the use of the propensity score matching method significantly reduced confounding bias by controlling plenty of confounders between the nonrandomized exposure and nonexposure groups in observational studies.^35^

### Limitations

This study has limitations. First, the strong association discovered in our study did not necessarily represent cause-effect due to the observational study design. Second, despite the adjustment for many underlying confounders, residual unidentified confounders may still exist. Third, since the baseline characteristics between participants included and excluded were different (eTable 15 in Supplement 1), the exclusion of 68 665 participants might cause selection bias. Fourth, White ethnicity accounted for 94.5% of the total study sample; hence, the representativeness of the sample was weakened, and decisions on the generalization of our findings should be cautious. Fifth, despite some evidence emphasizing the role of effective interventions in reducing subsequent dementia risk in patients with AF,^11^ we did not consider the information regarding treatment after AF onset due to lack of information on treatment, which warrants further investigation. Sixth, the identification of dementia cases in our study relied largely on the linkage from clinical records, which might induce bias because dementia could be underdiagnosed and underreported in the clinical settings.

## Conclusions

In this prospective cohort study of UKB participants, we discovered that earlier onset of AF was associated with an elevated risk of subsequent all-cause dementia, AD, and VD. The quantitative manifestation of the association between AF onset age and incident dementia highlights the importance of monitoring cognitive function among AF patients, especially those younger than 65 years at diagnosis.

## Notes

Supplement 1. **eTable 1.** *ICD-10* Codes Used to Ascertain All-Cause and Cause-Specific Dementia Cases **eTable 2.** Definition and Assessment of Covariates **eTable 3.** Baseline Characteristics of Participants With and Without a Diagnosis of Atrial Fibrillation (AF) After Propensity Score Matching (n=91 800) **eTable 4.** Associations of Atrial Fibrillation (AF) With Incident All-Cause Dementia, Alzheimer Disease, and Vascular Dementia Among Total Participants: Results From Cox Model Treating AF as a Time-Varying Variable (n=434 817) **eTable 5.** Associations of Age at Diagnosis of Atrial Fibrillation (AF) With Incident All-Cause Dementia, Alzheimer Disease, and Vascular Dementia Among AF Participants With Complete Covariates Data After Multiple Imputation (n=34 822) **eTable 6.** Associations of Age at Diagnosis of Atrial Fibrillation (AF) With Incident All-Cause Dementia, Alzheimer Disease, and Vascular Dementia Among AF Participants With Complete Covariates Data After Multiple Imputation: Results From Propensity Score Matching (n=104 463) **eTable 7.** Associations of Age at Diagnosis of Atrial Fibrillation (AF) With Incident All-Cause Dementia, Alzheimer Disease, and Vascular Dementia Among Participants With AF: Competing Risk Models (n=30 601) **eTable 8.** Associations of Atrial Fibrillation (AF) With Incident All-Cause Dementia, Alzheimer Disease, and Vascular Dementia Among Different Onset Age Groups After Propensity Score Matching: Competing Risk Models (n=91 800) **eTable 9.** Associations of Age at Diagnosis of Atrial Fibrillation (AF) With Incident All-Cause Dementia, Alzheimer Disease, and Vascular Dementia Among Participants With AF After Excluding Dementia Diagnosed Within 5 Years Since Baseline (n=30 554) **eTable 10.** Associations of Atrial Fibrillation (AF) With Incident All-Cause Dementia, Alzheimer Disease, and Vascular Dementia Among Different Onset Age Groups After Excluding Dementia Diagnosed Within 5 Years Since Baseline, Results From Propensity Score Matching Analyses (n=91 662) **eTable 11.** Associations of Age at Diagnosis of Atrial Fibrillation (AF) With Incident All-Cause Dementia, Alzheimer Disease, and Vascular Dementia Among Participants With AF After Excluding Participants With Baseline Age Younger Than 50 Years (n=29 109) **eTable 12.** Associations of Atrial Fibrillation (AF) With Incident All-Cause Dementia, Alzheimer Disease, and Vascular Dementia Among Different Onset Age Groups After Excluding Participants With Baseline Age Younger Than 50 Years, Results From Propensity Score Matching Analyses (n=87 315) **eTable 13.** Associations of Age at Diagnosis of Atrial Fibrillation (AF) With Incident All-Cause Dementia, Alzheimer Disease, and Vascular Dementia Among Participants With AF After Excluding Dementia Diagnosed Later Than December 31, 2019 (n=30 232) **eTable 14. **Associations of Atrial Fibrillation (AF) With Incident All-Cause Dementia, Alzheimer Disease, and Vascular Dementia Among Different Onset Age Groups After Excluding Dementia Diagnosed Later Than December 31, 2019: Results From Propensity Score Matching Analyses (n=90 693) **eTable 15.** Comparison of Baseline Characteristics Between Participants Included (n=433 746) and Excluded Due to History of Dementia or Stroke, Having Dementia Before Atrial Fibrillation (AF) Diagnosed During Follow-Up, or Missing Data of Covariates (n=68 665) **eFigure 1.** Subgroup Analyses to Identify Potential Modifying Effects From Covariates on the Association Between Atrial Fibrillation (AF) and Incident All-Cause Dementia by Using Cox Proportional Hazards Models (n=433 746) **eFigure 2.** Subgroup Analyses to Identify Potential Modifying Effects From Covariates on the Association Between Age at Diagnosis of Atrial Fibrillation (per 10 Years Decrease) and Incident All-Cause Dementia by Using Cox Proportional Hazards Models (n=30 601) **eFigure 3.** Subgroup Analyses to Identify Potential Modifying Effects From Covariates on the Association Between Atrial Fibrillation (AF) And Incident All-Cause Dementia in Participants With AF Diagnosed at Younger Than 65 Years and Their Controls by Using Cox Proportional Hazards Models (n=32 061) **eFigure 4.** Subgroup Analyses to Identify Potential Modifying Effects From Covariates on the Association Between Atrial Fibrillation (AF) and Incident All-Cause Dementia in Participants With AF Diagnosed at Age 65 to 74 Years and Their Controls by Using Cox Proportional Hazards Models (n=41 790) **eFigure 5.** Subgroup Analyses to Identify Potential Modifying Effects From Covariates on the Association Between Atrial Fibrillation (AF) and Incident All-Cause Dementia in Participants With AF Diagnosed at Age 75 Years and Older and Their Controls by Using Cox Proportional Hazards Models (n=17 949)

Supplement 2.
Data Sharing Statement
