# Supplementary material for: Age at Diagnosis of Atrial Fibrillation and Incident Dementia
Source: JAMA Netw Open. 2023 Nov 8;6(11):e2342744. doi: 10.1001/jamanetworkopen.2023.42744 (PMC10632957; doi:10.1001/jamanetworkopen.2023.42744)
Supplement: Supplement 1. — eTable 1. ICD-10 Codes Used to Ascertain All-Cause and Cause-Specific Dementia Cases eTable 2. Definition and Assessment of Covariates eTable 3. Baseline Characteristics of Participants With and Without a Diagnosis of Atrial Fibrillation (AF) After Propensity Score Matching (n=91 800) eTable 4. Associations of Atrial Fibrillation (AF) With Incident All-Cause Dementia, Alzheimer Disease, and Vascular Dementia Among Total Participants: Results From Cox Model Treating AF as a Time-Varying Variable (n=434 817) eTable 5. Associations of Age at Diagnosis of Atrial Fibrillation (AF) With Incident All-Cause Dementia, Alzheimer Disease, and Vascular Dementia Among AF Participants With Complete Covariates Data After Multiple Imputation (n=34 822) eTable 6. Associations of Age at Diagnosis of Atrial Fibrillation (AF) With Incident All-Cause Dementia, Alzheimer Disease, and Vascular Dementia Among AF Participants With Complete Covariates Data After Multiple Imputation: Results From Propensity Score Matching (n=104 463) eTable 7. Associations of Age at Diagnosis of Atrial Fibrillation (AF) With Incident All-Cause Dementia, Alzheimer Disease, and Vascular Dementia Among Participants With AF: Competing Risk Models (n=30 601) eTable 8. Associations of Atrial Fibrillation (AF) With Incident All-Cause Dementia, Alzheimer Disease, and Vascular Dementia Among Different Onset Age Groups After Propensity Score Matching: Competing Risk Models (n=91 800) eTable 9. Associations of Age at Diagnosis of Atrial Fibrillation (AF) With Incident All-Cause Dementia, Alzheimer Disease, and Vascular Dementia Among Participants With AF After Excluding Dementia Diagnosed Within 5 Years Since Baseline (n=30 554) eTable 10. Associations of Atrial Fibrillation (AF) With Incident All-Cause Dementia, Alzheimer Disease, and Vascular Dementia Among Different Onset Age Groups After Excluding Dementia Diagnosed Within 5 Years Since Baseline, Results From Propensity Score Matching Analyses (n=91 662) eTable 11. [file jamanetwopen-e2342744-s001.pdf]

Supplemental Online Content

Zhang W, Liang J, Li C, et al. Age at diagnosis of atrial fibrillation and incident dementia. *JAMA Netw Open*. 2023;6(11):e2342744. doi:10.1001/jamanetworkopen.2023.42744

eTable 1. *ICD-10* Codes Used to Ascertain All-Cause and Cause-Specific Dementia Cases

eTable 2. Definition and Assessment of Covariates

eTable 3. Baseline Characteristics of Participants With and Without a Diagnosis of Atrial Fibrillation (AF) After Propensity Score Matching (n=91 800)

eTable 4. Associations of Atrial Fibrillation (AF) With Incident All-Cause Dementia, Alzheimer Disease, and Vascular Dementia Among Total Participants: Results From Cox Model Treating AF as a Time-Varying Variable (n=434 817)

eTable 5. Associations of Age at Diagnosis of Atrial Fibrillation (AF) With Incident All-Cause Dementia, Alzheimer Disease, and Vascular Dementia Among AF Participants With Complete Covariates Data After Multiple Imputation (n=34 822)

eTable 6. Associations of Age at Diagnosis of Atrial Fibrillation (AF) With Incident All-Cause Dementia, Alzheimer Disease, and Vascular Dementia Among AF Participants With Complete Covariates Data After Multiple Imputation: Results From Propensity Score Matching (n=104 463)

eTable 7. Associations of Age at Diagnosis of Atrial Fibrillation (AF) With Incident All-Cause Dementia, Alzheimer Disease, and Vascular Dementia Among Participants With AF: Competing Risk Models (n=30 601)

eTable 8. Associations of Atrial Fibrillation (AF) With Incident All-Cause Dementia, Alzheimer Disease, and Vascular Dementia Among Different Onset Age Groups After Propensity Score Matching: Competing Risk Models (n=91 800)

eTable 9. Associations of Age at Diagnosis of Atrial Fibrillation (AF) With Incident All-Cause Dementia, Alzheimer Disease, and Vascular Dementia Among Participants With AF After Excluding Dementia Diagnosed Within 5 Years Since Baseline (n=30 554)

eTable 10. Associations of Atrial Fibrillation (AF) With Incident All-Cause Dementia, Alzheimer Disease, and Vascular Dementia Among Different Onset Age Groups After Excluding Dementia Diagnosed Within 5 Years Since Baseline, Results From Propensity Score Matching Analyses (n=91 662)

eTable 11. Associations of Age at Diagnosis of Atrial Fibrillation (AF) With Incident All-Cause Dementia, Alzheimer Disease, and Vascular Dementia Among Participants With AF After Excluding Participants With Baseline Age Younger Than 50 Years (n=29 109)

eTable 12. Associations of Atrial Fibrillation (AF) With Incident All-Cause Dementia, Alzheimer Disease, and Vascular Dementia Among Different Onset Age Groups After Excluding Participants

© 2023 Zhang W et al. *JAMA Network Open.*

With Baseline Age Younger Than 50 Years, Results From Propensity Score Matching Analyses (n=87 315)

eTable 13. Associations of Age at Diagnosis of Atrial Fibrillation (AF) With Incident All-Cause Dementia, Alzheimer Disease, and Vascular Dementia Among Participants With AF After Excluding Dementia Diagnosed Later Than December 31, 2019 (n=30 232)

eTable 14. Associations of Atrial Fibrillation (AF) With Incident All-Cause Dementia, Alzheimer Disease, and Vascular Dementia Among Different Onset Age Groups After Excluding Dementia Diagnosed Later Than December 31, 2019: Results From Propensity Score Matching Analyses (n=90 693)

eTable 15. Comparison of Baseline Characteristics Between Participants Included (n=433 746) and Excluded Due to History of Dementia or Stroke, Having Dementia Before Atrial Fibrillation (AF) Diagnosed During Follow-Up, or Missing Data of Covariates (n=68 665)

eFigure 1. Subgroup Analyses to Identify Potential Modifying Effects From Covariates on the Association Between Atrial Fibrillation (AF) and Incident All-Cause Dementia by Using Cox Proportional Hazards Models (n=433 746)

eFigure 2. Subgroup Analyses to Identify Potential Modifying Effects From Covariates on the Association Between Age at Diagnosis of Atrial Fibrillation (per 10 Years Decrease) and Incident All-Cause Dementia by Using Cox Proportional Hazards Models (n=30 601)

eFigure 3. Subgroup Analyses to Identify Potential Modifying Effects From Covariates on the Association Between Atrial Fibrillation (AF) And Incident All-Cause Dementia in Participants With AF Diagnosed at Younger Than 65 Years and Their Controls by Using Cox Proportional Hazards Models (n=32 061)

eFigure 4. Subgroup Analyses to Identify Potential Modifying Effects From Covariates on the Association Between Atrial Fibrillation (AF) and Incident All-Cause Dementia in Participants With AF Diagnosed at Age 65 to 74 Years and Their Controls by Using Cox Proportional Hazards Models (n=41 790)

eFigure 5. Subgroup Analyses to Identify Potential Modifying Effects From Covariates on the Association Between Atrial Fibrillation (AF) and Incident All-Cause Dementia in Participants With AF Diagnosed at Age 75 Years and Older and Their Controls by Using Cox Proportional Hazards Models (n=17 949)

This supplemental material has been provided by the authors to give readers additional information about their work.

© 2023 Zhang W et al. *JAMA Network Open.*

eTable 1. *ICD-10* Codes Used to Ascertain All-Cause and Cause-Specific Dementia Cases

ICD 10 Code	Code description	Alzheimer's disease	Vascular dementia	Frontotemporal dementia	All-cause dementia
A81.0	Sporadic Creutzfeldt-Jakob disease	0	0	0	1
F00	Dementia in Alzheimer's disease	1	0	0	1
F00.0	Dementia in Alzheimer's disease with early onset	1	0	0	1
F00.1	Dementia in Alzheimer's disease with late onset	1	0	0	1
F00.2	Dementia in Alzheimer's disease, atypical or mixed type	1	0	0	1
F00.9	Dementia in Alzheimer's disease, unspecified	1	0	0	1
F01	Vascular dementia	0	1	0	1
F01.0	Vascular dementia of acute onset	0	1	0	1
F01.1	Multi-infarct dementia	0	1	0	1
F01.2	Subcortical vascular dementia	0	1	0	1
F01.3	Mixed cortical and sub-cortical vascular dementia	0	1	0	1
F01.8	Other vascular dementia	0	1	0	1
F01.9	Vascular dementia, unspecified	0	1	0	1
F02	Dementia in other diseases classified elsewhere	0	0	0	1
F02.0	Dementia in Picks disease	0	0	1	1
F02.1	Dementia in Creutzfeldt-Jacob disease	0	0	0	1
F02.2	Dementia in Huntington's disease	0	0	0	1
F02.3	Dementia in Parkinson's disease	0	0	0	1
F02.4	Dementia in HIV disease	0	0	0	1

© 2023 Zhang W et al. *JAMA Network Open.*

ICD 10 Code	Code description	Alzheimer’s disease	Vascular dementia	Frontotemporal dementia	All-cause dementia
F02.8	Dementia in other specified diseases classified elsewhere	0	0	0	1
F03	Unspecified dementia	0	0	0	1
F05.1	Delirium superimposed on dementia	0	0	0	1
F10.6	Mental and behavioral disorders due to use of alcohol - amnesic syndrome	0	0	0	1
G30	Alzheimer’s disease	1	0	0	1
G30.0	Alzheimer’s disease with early onset	1	0	0	1
G30.1	Alzheimer’s disease with late onset	1	0	0	1
G30.8	Other Alzheimer's disease	1	0	0	1
G30.9	Alzheimer's disease unspecified	1	0	0	1
G31.0	Circumscribed brain atrophy	0	0	1	1
G31.1	Senile degeneration of brain	0	0	0	1
G31.8	Other specified degenerative diseases of nervous system	0	0	0	1
I67.3	Binswanger's disease	0	1	0	1

1=yes, 0=no.

© 2023 Zhang W et al. *JAMA Network Open.*

eTable 2. Definition and Assessment of Covariates

Covariates	Definition	Assessment	UK biobank Data-Field ID
Age (years)	Age in years	Difference between date attended baseline assessment and date of birth recorded by NHS	21003
Sex	Men, Women	NHS derived and/or touchscreen questionnaire	31
Ethnicity	White, Non-white (Mixed, Asian, Black, Chinese, Other)	Touchscreen questionnaire: "What is your ethnic group?"	21000
Education	Higher education (college or university degree, other professional qualifications), other than higher education	Touchscreen questionnaire: "Which of the following qualifications do you have?"	6138
Current smoking	Yes, No	Touchscreen questionnaire: "Do you smoke tobacco now?" and "In the past, how often have you smoked tobacco?"	20116
Current drinking	At least once per week, less than once per week	Touchscreen questionnaire: "About how often do you drink alcohol?"	1558
Physical activity	Attending moderate or vigorous physical activity 10+ minutes at least twice per week, less than twice per week	Touchscreen questionnaire: "In a typical WEEK, on how many days did you do 10 minutes or more of moderate physical activities like carrying light loads, cycling at normal pace? (Do not include walking);<br>In a typical WEEK, how many days did you do 10 minutes or more of vigorous physical activity? (These are activities that make you sweat or breathe hard such as fast cycling, aerobics, heavy lifting)"	884, 904

© 2023 Zhang W et al. *JAMA Network Open.*

Covariates	Definition	Assessment	UK biobank Data-Field ID
Depressed mood	Yes (nearly every day or more than half the days), No (not at all or several days)	Touchscreen questionnaire: "Over the past two weeks, how often have you felt down, depressed or hopeless?"	2050
Hypertension	Yes, No	Touchscreen questionnaire and verbal interview: self-reported hypertension or anti-hypertensive medication use;<br>Average SBP/DBP ≥ 140/90 mmHg at baseline	6150, 20002, 6177, 4079, 4080, 93, 94
Diabetes	Yes, No	Touchscreen questionnaire and verbal interview: self-reported diabetes (diabetes, type 1 diabetes or type 2 diabetes) or medication use for lowering blood glucose;<br>Plasma HbA1c ≥ 48 mmol/mol (6.5%)	2443, 20002, 6153, 6177, 30750, 20003
Coronary heart disease	Yes, No	Touchscreen questionnaire and verbal interview: angina, heart attack/myocardial infarction	6150, 20002
BMI (kg/m^2)	Continuous	Physical examination: body mass index, calculated from height and weight measured during the initial Assessment Centre visit	21001
SBP (mmHg)	Continuous	Physical measures: systolic blood pressure, measured twice by an automated device named Omron. A manual sphygmometer was used if the standard automated device could not be employed.	4080, 93
DBP (mmHg)	Continuous	Physical measures: systolic blood pressure, measured twice by an automated device named Omron. A manual sphygmometer was used if the standard automated device could not be employed.	4079, 94
HbA_{1c} (mmol/mol)	Continuous	Blood biochemistry: glycated haemoglobin, measured by HPLC analysis on a Bio-Rad VARIANT II Turbo	30750

© 2023 Zhang W et al. *JAMA Network Open.*

Covariates	Definition	Assessment	UK biobank Data-Field ID
LDL-C (mmol/L)	Continuous	Blood biochemistry: low-density lipoprotein cholesterol, measured by enzymatic protective selection analysis on a Beckman Coulter AU5800	30780
Use of antihypertensive drugs	Yes, No	Touchscreen questionnaire: self-reported blood pressure medication	6177, 6153
Use of antihyperglycemic drugs	Yes, No	Touchscreen questionnaire and verbal interview: self-reported insulin use and antihyperglycemic drugs	6177, 6153, 20003
Use of statin	Yes, No	Verbal interview: self-reported statin use	20003
ApoE4 carrier	Yes, No	Genomics	affy160203316 affy160203324

BMI, body mass index; SBP, systolic blood pressure; DBP, diastolic blood pressure; HbA1c, glycated hemoglobin; LDL-C, low-density lipoprotein cholesterol.

© 2023 Zhang W et al. *JAMA Network Open.*

eTable 3. Baseline Characteristics of Participants With and Without a Diagnosis of Atrial Fibrillation (AF) After Propensity Score Matching (n=91 800)

Characteristics	AF	Non-AF	P-value[a]
**< 65 years (n=32 061)**			
Age, mean (SD), y	57.9 (7.1)	57.9 (7.9)	.94
Female, No. (%)	3411 (31.9)	6870 (32.1)	.69
White, No. (%)	10 315 (96.5)	20 631 (96.5)	.98
Higher education, No. (%)	4931 (46.1)	9926 (46.4)	.61
BMI, mean (SD), kg/m2	29.1 (5.6)	29.1 (5.4)	.80
HbA_{1c}, mean (SD), mmol/mol	37.6 (8.7)	37.4 (8.3)	.13
SBP, mean (SD), mmHg	137.9 (18.8)	137.9 (17.2)	.85
DBP, mean (SD), mmHg	83.0 (11.1)	82.9 (10.0)	.36
LDL-C, mean (SD), mmol/L	3.3 (0.9)	3.3 (0.9)	.97
Current smoking, No. (%)	1104 (10.3)	2171 (10.2)	.63
Current drinking, No. (%)	7505 (70.2)	14 970 (70.0)	.73
Exercise, No. (%)	8110 (75.9)	16 205 (75.8)	.89
Depressed mood, No. (%)	582 (5.5)	1141 (5.3)	.69
Diabetes, No. (%)	1227 (11.5)	2460 (11.5)	.94
Hypertension, No. (%)	7089 (66.3)	14 203 (66.5)	.83
Coronary heart disease, No. (%)	1558 (14.6)	3137 (14.7)	.82
Use of antihypertensive drugs, No. (%)	4119 (38.5)	6950 (32.5)	<.001
Use of antihyperglycemic drugs, No. (%)	726 (6.8)	1549 (7.3)	.14
Use of statin, No. (%)	3163 (29.6)	6321 (29.6)	.97
ApoE4 carrier, No. (%)	2488 (23.3)	5033 (23.6)	.60
**65-74 years (n=41 790)**			
Age, mean (SD), y	63.6 (3.8)	63.9 (4.5)	<.001
Female, No. (%)	5263 (37.8)	11 052 (39.7)	<.001
White, No. (%)	13 532 (97.1)	27 102 (97.3)	.42
Higher education, No. (%)	5581 (40.1)	11 158 (40.1)	.98
BMI, mean (SD), kg/m2	29.0 (5.3)	28.8 (5.0)	<.001

© 2023 Zhang W et al. *JAMA Network Open.*

HbA_{1c}, mean (SD), mmol/mol	37.9 (7.8)	37.7 (7.3)	.04
SBP, mean (SD), mmHg	144.0 (19.1)	144.4 (18.1)	.07
DBP, mean (SD), mmHg	82.9 (10.5)	83.1 (9.9)	.02
LDL-C, mean (SD), mmol/L	3.4 (0.9)	3.4 (0.9)	<.001
Current Smoking, No. (%)	1424 (10.2)	2819 (10.1)	.74
Current drinking, No. (%)	9930 (71.3)	19 900 (71.4)	.76
Exercise, No. (%)	10 724 (77.0)	21 450 (77.0)	.99
Depressed mood, No. (%)	591 (4.2)	1192 (4.3)	.86
Diabetes, No. (%)	1606 (11.5)	2930 (10.5)	.002
Hypertension, No. (%)	10 568 (75.9)	21 125 (75.8)	.93
Coronary heart disease, No. (%)	1914 (13.7)	3311 (11.9)	<.001
Use of antihypertensive drugs, No. (%)	5988 (43.0)	9882 (35.5)	<.001
Use of antihyperglycemic drugs, No. (%)	995 (7.1)	1774 (6.4)	.003
Use of statin, No. (%)	4208 (30.2)	7960(28.6)	<.001
ApoE4 carrier, No. (%)	3308 (23.8)	6357 (22.8)	.03
**≥75 years (n=17 949)**			
Age, mean (SD), y	67.4 (2.0)	67.4 (2.1)	.09
Female, No. (%)	2517 (42.1)	5201 (43.5)	.08
White, No. (%)	5848 (97.7)	11 701 (97.8)	.86
Higher education, No. (%)	2179 (36.4)	4410 (36.9)	.57
BMI, mean (SD), kg/m2	28.4 (4.8)	28.3 (4.6)	.03
HbA_{1c}, mean (SD), mmol/mol	37.9 (7.4)	37.9 (7.2)	.98
SBP, mean (SD), mmHg	147.6 (19.1)	147.4 (18.6)	.59
DBP, mean (SD), mmHg	82.0 (10.0)	82.0 (9.8)	.82
LDL-C, mean (SD), mmol/L	3.3 (0.9)	3.4 (0.9)	.01
Current smoking, No. (%)	499 (8.3)	955 (8.0)	.41
Current drinking, No. (%)	4107 (68.6)	8200 (68.5)	.87
Exercise, No. (%)	4735 (79.1)	9438 (78.9)	.68
Depressed mood, No. (%)	183 (3.1)	351 (2.9)	.64

© 2023 Zhang W et al. *JAMA Network Open.*

Diabetes, No. (%)	710 (11.9)	1300 (10.9)	.05
Hypertension, No. (%)	4804 (80.3)	9516 (79.5)	.23
Coronary heart disease, No. (%)	773 (12.9)	1449 (12.1)	.12
Use of antihypertensive drugs, No. (%)	2719 (45.5)	4676 (39.1)	<.001
Use of antihyperglycemic drugs, No. (%)	418 (7.0)	768 (6.4)	.15
Use of statin, No. (%)	1989 (33.2)	3788 (31.7)	.03
ApoE4 carrier, No. (%)	1365 (22.8)	2727 (22.8)	.97

Abbreviations: BMI, body mass index; HbA_{1c}, glycated hemoglobin; SBP, systolic blood pressure; DBP, diastolic blood pressure; LDL-C, low-density lipoprotein cholesterol.
The results are presented as mean (SD) or No. (%).
[a]Calculated by using a *t* test, or chi-square test.

© 2023 Zhang W et al. *JAMA Network Open.*

eTable 4. Associations of Atrial Fibrillation (AF) With Incident All-Cause Dementia, Alzheimer's Disease, and Vascular Dementia Among Total Participants: Results From Cox Model Treating AF as a Time-Varying Variable (n=434 817)

Outcome	HR (95% CI) AF vs. Non-AF	P-value
All-cause dementia		
Model 1[a]	2.34 (2.18-2.50)	<.001
Model 2[b]	2.21 (2.06-2.37)	<.001
Alzheimer's disease		
Model 1[a]	1.68 (1.50-1.89)	<.001
Model 2[b]	1.64 (1.46-1.85)	<.001
Vascular dementia		
Model 1[a]	3.61 (3.16-4.13)	<.001
Model 2[b]	3.19 (2.78-3.65)	<.001

Abbreviations: HR, hazard ratio; CI, confidence interval.

[a]Adjusted for age, sex, ethnicity, education and birth cohort (5-year groups).

[b]Further adjusted for baseline body mass index, low-density lipoprotein cholesterol, current smoking, current drinking, exercise, depressed mood, hypertension, diabetes, coronary heart disease, use of antihypertensive drugs, use of antihyperglycemic drugs, use of statin, and ApoE4 status.

© 2023 Zhang W et al. *JAMA Network Open.*

eTable 5. Associations of Age at Diagnosis of Atrial Fibrillation (AF) With Incident All-Cause Dementia, Alzheimer Disease, and Vascular Dementia Among AF Participants With Complete Covariates Data After Multiple Imputation (n=34 822)

Outcome	HR (95% CI)[a]	P-value
All-cause dementia		
≥75 years (n=6840)	Reference	NA
65-74 years (n=15 833)	1.50 (1.30-1.73)	<.001
<65 years (n=12 149)	1.80 (1.52-2.13)	<.001
Per 10-year decrease	1.25 (1.18-1.33)	<.001
Alzheimer's disease		
≥75 years (n=6840)	Reference	NA
65-74 years (n=15 833)	1.47 (1.16-1.86)	.002
<65 years (n=12 149)	1.82 (1.37-2.41)	<.001
Per 10-year decrease	1.28 (1.16-1.42)	<.001
Vascular dementia		
≥75 years (n=6840)	Reference	NA
65-74 years (n=15 833)	1.93 (1.48-2.50)	<.001
<65 years (n=12 149)	2.07 (1.51-2.84)	<.001
Per 10-year decrease	1.35 (1.22-1.50)	<.001

Abbreviations: HR, hazard ratio; CI, confidence interval; NA, not applicable.

[a]Adjusted for age, sex, race, education, baseline body mass index, low-density lipoprotein cholesterol, current smoking, current drinking, exercise, depressed mood, hypertension, diabetes, coronary heart disease, use of antihypertensive drugs, use of antihyperglycemic drugs, use of statin, and ApoE4 status.

© 2023 Zhang W et al. *JAMA Network Open.*

eTable 6. Associations of Age at Diagnosis of Atrial Fibrillation (AF) With Incident All-Cause Dementia, Alzheimer Disease, and Vascular Dementia Among AF Participants With Complete Covariates Data After Multiple Imputation: Results From Propensity Score Matching (n=104 463)

Outcome	HR (95% CI)[a]	P-value
All-cause dementia		
≥75 years (n=20 520)	1.10 (0.96-1.26)	.19
65-74 years (n=47 496)	1.57 (1.41-1.76)	<.001
<65 years (n=36 447)	1.86 (1.60-2.18)	<.001
Alzheimer’s disease		
≥75 years (n=20 520)	0.85 (0.69-1.06)	.15
65-74 years (n=47 496)	1.17 (0.99-1.40)	.07
<65 years (n=36 447)	1.53 (1.18-1.97)	.001
Vascular dementia		
≥75 years (n=20 520)	1.35 (1.02-1.78)	.03
65-74 years (n=47 496)	2.31 (1.89-2.83)	<.001
<65 years (n=36 447)	2.49 (1.85-3.36)	<.001

Abbreviations: HR, hazard ratio; CI, confidence interval.

[a]Adjusted for age, sex, race, education, baseline body mass index, low-density lipoprotein cholesterol, current smoking, current drinking, exercise, depressed mood, hypertension, diabetes, coronary heart disease, use of antihypertensive drugs, use of antihyperglycemic drugs, use of statin, and ApoE4 status.

© 2023 Zhang W et al. *JAMA Network Open.*

eTable 7. Associations of Age at Diagnosis of Atrial Fibrillation (AF) With Incident All-Cause Dementia, Alzheimer Disease, and Vascular Dementia Among Participants With AF: Competing Risk Models (n=30 601)

Outcome	HR (95% CI)[a]	P-value
All-cause dementia		
≥75 years (n=5984)	Reference	NA
65-74 years (n=13 930)	1.25 (1.08-1.46)	.003
<65 years (n=10 687)	1.47 (1.22-1.77)	<.001
Per 10 years decrease	1.15 (1.07-1.23)	<.001
Alzheimer's disease		
≥75 years (n=5984)	Reference	NA
65-74 years (n=13 930)	1.24 (0.96-1.60)	.10
<65 years (n=10 687)	1.49 (1.09-2.03)	.01
Per 10 years decrease	1.18 (1.05-1.34)	.007
Vascular dementia		
≥75 years (n=5984)	Reference	NA
65-74 years (n=13 930)	1.72 (1.30-2.29)	<.001
<65 years (n=10 687)	1.77 (1.25-2.52)	.002
Per 10 years decrease	1.24 (1.09-1.41)	<.001

Abbreviations: HR, hazard ratio; CI, confidence interval.

[a]Adjusted for age, sex, race, education, baseline body mass index, low-density lipoprotein cholesterol, current smoking, current drinking, exercise, depressed mood, hypertension, diabetes, coronary heart disease, use of antihypertensive drugs, use of antihyperglycemic drugs, use of statin, and ApoE4 status.

eTable 8. Associations of Atrial Fibrillation (AF) With Incident All-Cause Dementia, Alzheimer Disease, and Vascular Dementia Among Different Onset Age Groups After Propensity Score Matching: Competing Risk Models (n=91 800)

Outcome	HR (95% CI)[a] AF vs. non-AF	P-value
All-cause Dementia		
≥75 years (n=17 949)	1.15 (1.00-1.33)	.05
65-74 years (n=41 790)	1.36 (1.21-1.52)	<.001
<65 years (n=32 061)	1.66 (1.41-1.97)	<.001
Alzheimer's Disease		
≥75 years (n=17 949)	0.87 (0.69-1.09)	.22
65-74 years (n=41 790)	1.01 (0.84-1.22)	.93
<65 years (n=32 061)	1.26 (0.95-1.66)	.11
Vascular Dementia		
≥75 years (n=17 949)	1.31 (0.97-1.76)	.08
65-74 years (n=41 790)	2.09 (1.69-2.59)	<.001
<65 years (n=32 061)	2.78 (1.98-3.91)	<.001

Abbreviations: HR, hazard ratio; CI, confidence interval.

[a]Adjusted for age, sex, race, education, baseline body mass index, low-density lipoprotein cholesterol, current smoking, current drinking, exercise, depressed mood, hypertension, diabetes, coronary heart disease, use of antihypertensive drugs, use of antihyperglycemic drugs, use of statin, and ApoE4 status.

© 2023 Zhang W et al. *JAMA Network Open.*

eTable 9. Associations of Age at Diagnosis of Atrial Fibrillation (AF) With Incident All-Cause Dementia, Alzheimer Disease, and Vascular Dementia Among Participants With AF After Excluding Dementia Diagnosed Within 5 Years Since Baseline (n=30 554)

Outcome	HR (95% CI)[a]	P-value
All-cause dementia		
≥75 years (n=5984)	Reference	NA
65-74 years (n=13 904)	1.38 (1.18-1.61)	<.001
<65 years (n=10 666)	1.59 (1.32-1.92)	<.001
Per 10 years decrease	1.20 (1.12-1.29)	<.001
Alzheimer's disease		
≥75 years (n=5984)	Reference	NA
65-74 years (n=13 904)	1.38 (1.06-1.78)	.02
<65 years (n=10 666)	1.60 (1.17-2.20)	.004
Per 10 years decrease	1.24 (1.10-1.40)	<.001
Vascular dementia		
≥75 years (n=5984)	Reference	NA
65-74 years (n=13 904)	1.94 (1.46-2.58)	<.001
<65 years (n=10 666)	1.90 (1.32-2.73)	<.001
Per 10 years decrease	1.31 (1.15-1.48)	<.001

Abbreviations: HR, hazard ratio; CI, confidence interval; NA, not applicable.

[a]Adjusted for age, sex, race, education, baseline body mass index, low-density lipoprotein cholesterol, current smoking, current drinking, exercise, depressed mood, hypertension, diabetes, coronary heart disease, use of antihypertensive drugs, use of antihyperglycemic drugs, use of statin, and ApoE4 status.

© 2023 Zhang W et al. *JAMA Network Open.*

eTable 10. Associations of Atrial Fibrillation (AF) With Incident All-Cause Dementia, Alzheimer Disease, and Vascular Dementia Among Different Onset Age Groups After Excluding Dementia Diagnosed Within 5 Years Since Baseline, Results From Propensity Score Matching Analyses (n=91 662)

Outcome	HR (95% CI)[a] AF vs. non-AF	P-value
All-cause Dementia		
≥75 years (n=17 952)	1.28 (1.10-1.49)	.001
65-74 years (n=41 712)	1.68 (1.49-1.89)	<.001
<65 years (n=31 998)	1.83 (1.53-2.18)	<.001
Alzheimer's Disease		
≥75 years (n=17 952)	0.93 (0.73-1.17)	.52
65-74 years (n=41 712)	1.27 (1.04-1.55)	.02
<65 years (n=31 998)	1.41 (1.05-1.88)	.02
Vascular Dementia		
≥75 years (n=17 952)	1.50 (1.09-2.05)	.01
65-74 years (n=41 712)	2.47 (1.97-3.10)	<.001
<65 years (n=31 998)	2.67 (1.86-3.85)	<.001

Abbreviations: HR, hazard ratio; CI, confidence interval.

[a]Adjusted for age, sex, race, education, baseline body mass index, low-density lipoprotein cholesterol, current smoking, current drinking, exercise, depressed mood, hypertension, diabetes, coronary heart disease, use of antihypertensive drugs, use of antihyperglycemic drugs, use of statin, and ApoE4 status.

© 2023 Zhang W et al. *JAMA Network Open.*

eTable 11. Associations of Age at Diagnosis of Atrial Fibrillation (AF) With Incident All-Cause Dementia, Alzheimer Disease, and Vascular Dementia Among Participants With AF After Excluding Participants With Baseline Age Younger Than 50 Years (n=29 109)

Outcome	HR (95% CI)[a]	P-value
All-cause dementia		
≥75 years (n=5984)	Reference	NA
65-74 years (n=13 930)	1.45 (1.25-1.69)	<.001
<65 years (n=9195)	1.74 (1.45-2.09)	<.001
Per 10 years decrease	1.24 (1.16-1.32)	<.001
Alzheimer's disease		
≥75 years (n=5984)	Reference	NA
65-74 years (n=13 930)	1.43 (1.11-1.85)	.006
<65 years (n=9195)	1.75 (1.29-2.38)	<.001
Per 10 years decrease	1.27 (1.13-1.42)	<.001
Vascular dementia		
≥75 years (n=5984)	Reference	NA
65-74 years (n=13 930)	2.04 (1.54-2.71)	<.001
<65 years (n=9195)	2.17 (1.54-3.07)	<.001
Per 10 years decrease	1.35 (1.20-1.51)	<.001

Abbreviations: HR, hazard ratio; CI, confidence interval; NA, not applicable.

[a]Adjusted for age, sex, race, education, baseline body mass index, low-density lipoprotein cholesterol, current smoking, current drinking, exercise, depressed mood, hypertension, diabetes, coronary heart disease, use of antihypertensive drugs, use of antihyperglycemic drugs, use of statin, and ApoE4 status.

© 2023 Zhang W et al. *JAMA Network Open.*

eTable 12. Associations of Atrial Fibrillation (AF) With Incident All-Cause Dementia, Alzheimer Disease, and Vascular Dementia Among Different Onset Age Groups After Excluding Participants With Baseline Age Younger Than 50 Years, Results From Propensity Score Matching Analyses (n=87 315)

Outcome	HR (95% CI)[a] AF vs. non-AF	P-value
All-cause Dementia		
≥75 years (n=17 949)	1.08 (0.93-1.25)	.32
65-74 years (n=41 790)	1.57 (1.40-1.77)	<.001
<65 years (n=27 576)	1.75 (1.47-2.07)	<.001
Alzheimer's Disease		
≥75 years (n=17 949)	0.77 (0.61-0.97)	.03
65-74 years (n=41 790)	1.18 (0.97-1.42)	.10
<65 years (n=27 576)	1.28 (0.97-1.69)	.09
Vascular Dementia		
≥75 years (n=17 949)	1.42 (1.05-1.92)	.03
65-74 years (n=41 790)	2.40 (1.93-2.99)	<.001
<65 years (n=27 576)	2.59 (1.87-3.61)	<.001

Abbreviations: HR, hazard ratio; CI, confidence interval.

[a]Adjusted for age, sex, race, education, baseline body mass index, low-density lipoprotein cholesterol, current smoking, current drinking, exercise, depressed mood, hypertension, diabetes, coronary heart disease, use of antihypertensive drugs, use of antihyperglycemic drugs, use of statin, and ApoE4 status.

eTable 13. Associations of Age at Diagnosis of Atrial Fibrillation (AF) With Incident All-Cause Dementia, Alzheimer Disease, and Vascular Dementia Among Participants With AF After Excluding Dementia Diagnosed Later Than December 31, 2019 (n=30 232)

Outcome	HR (95% CI)[a]	P-value
All-cause dementia		
≥75 years (n=5825)	Reference	NA
65-74 years (n=13 782)	2.24 (1.82-2.75)	<.001
<65 years (n=10 625)	2.89 (2.29-3.65)	<.001
Per 10 years decrease	1.40 (1.31-1.50)	<.001
Alzheimer's disease		
≥75 years (n=5825)	Reference	NA
65-74 years (n=13 782)	2.10 (1.52-2.92)	<.001
<65 years (n=10 625)	2.67 (1.83-3.90)	<.001
Per 10 years decrease	1.44 (1.28-1.62)	<.001
Vascular dementia		
≥75 years (n=5825)	Reference	NA
65-74 years (n=13 782)	3.42 (2.35-4.98)	<.001
<65 years (n=10 625)	3.80 (2.47-5.82)	<.001
Per 10 years decrease	1.49 (1.32-1.68)	<.001

Abbreviations: HR, hazard ratio; CI, confidence interval; NA, not applicable.

[a]Adjusted for age, sex, race, education, baseline body mass index, low-density lipoprotein cholesterol, current smoking, current drinking, exercise, depressed mood, hypertension, diabetes, coronary heart disease, use of antihypertensive drugs, use of antihyperglycemic drugs, use of statin, and ApoE4 status.

© 2023 Zhang W et al. *JAMA Network Open.*

eTable 14. Associations of Atrial Fibrillation (AF) With Incident All-Cause Dementia, Alzheimer Disease, and Vascular Dementia Among Different Onset Age Groups After Excluding Dementia Diagnosed Later Than December 31, 2019: Results From Propensity Score Matching Analyses (n=90 693)

Outcome	HR (95% CI)[a] AF vs. non-AF	P-value
All-cause Dementia		
≥75 years (n=17 472)	0.66 (0.54-0.81)	<.001
65-74 years (n=41 346)	1.45 (1.27-1.67)	<.001
<65 years (n=31 875)	1.52 (1.26-1.83)	<.001
Alzheimer's Disease		
≥75 years (n=17 472)	0.53 (0.39-0.73)	<.001
65-74 years (n=41 346)	1.07 (0.86-1.33)	.56
<65 years (n=31 875)	1.15 (0.85-1.56)	.36
Vascular Dementia		
≥75 years (n=17 472)	0.66 (0.45-0.98)	.04
65-74 years (n=41 346)	2.59 (2.01-3.33)	<.001
<65 years (n=31 875)	2.42 (1.70-3.46)	<.001

Abbreviations: HR, hazard ratio; CI, confidence interval.

[a]Adjusted for age, sex, race, education, baseline body mass index, low-density lipoprotein cholesterol, current smoking, current drinking, exercise, depressed mood, hypertension, diabetes, coronary heart disease, use of antihypertensive drugs, use of antihyperglycemic drugs, use of statin, and ApoE4 status.

© 2023 Zhang W et al. *JAMA Network Open.*

eTable 15. Comparison of Baseline Characteristics Between Participants Included (n=433 746) and Excluded Due to History of Dementia or Stroke, Having Dementia Before Atrial Fibrillation (AF) Diagnosed During Follow-Up, or Missing Data of Covariates (n=68 665)

**Characteristics**	**Participants included (n=433 746)**	**Participants excluded (n=68 665)**	**P-value[a]**
Age, mean (SD), y	56.9 (8.1)	57.6 (8.1)	<.001
Female, No. (%)	236 253 (54.5)	37 072 (54.0)	.02
White, No. (%)	409 990 (94.5)	62 053 (90.4)	<.001
Higher education, No. (%)	204 503 (47.2)	29 041 (42.3)	<.001
BMI, mean (SD), kg/m2	27.4 (4.7)	27.8 (5.1)	<.001
HbA_{1c}, mean (SD), mmol/mol	36.0 (6.6)	37.3 (8.6)	<.001
SBP, mean (SD), mmHg	137.8 (18.6)	138.3 (18.9)	<.001
DBP, mean (SD), mmHg	82.3 (10.1)	82.1 (10.3)	<.001
LDL-C, mean (SD), mmol/L	3.6 (0.9)	3.4 (0.9)	<.001
Current smoking, No. (%)	45 107 (10.4)	7855 (11.4)	<.001
Current drinking, No. (%)	301 649 (69.6)	44 797 (65.2)	<.001
Exercise, No. (%)	339 206 (78.2)	51 542 (75.1)	<.001
Depressed mood, No. (%)	20 284 (4.7)	4010 (5.8)	<.001
Diabetes, No. (%)	25 462 (5.9)	5264 (7.7)	<.001
Hypertension, No. (%)	237 277 (54.7)	39 747 (57.9)	<.001
Coronary heart disease, No. (%)	18 812 (4.3)	4473 (6.5)	<.001
Use of antihypertensive drugs, No. (%)	86 893 (20.0)	17 094 (24.9)	<.001
Use of antihyperglycemic drugs, No. (%)	14 886 (3.4)	3324 (4.8)	<.001
Use of statin, No. (%)	63 406 (14.6)	14 615 (21.3)	<.001
ApoE4 carrier, No. (%)	103 862 (24.0)	13 975 (20.4)	<.001

Abbreviations: BMI, body mass index; SBP, systolic blood pressure; DBP, diastolic blood pressure; HbA_{1c}, glycated hemoglobin; LDL-C, low-density lipoprotein cholesterol.
The results are presented as mean (SD) or No. (%).
[a]Calculated by using a *t* test, or chi-square test.

© 2023 Zhang W et al. *JAMA Network Open.*

eFigure 1. Subgroup Analyses to Identify Potential Modifying Effects From Covariates on the Association Between Atrial Fibrillation (AF) and Incident All-Cause Dementia by Using Cox Proportional Hazards Models (n=433 746)

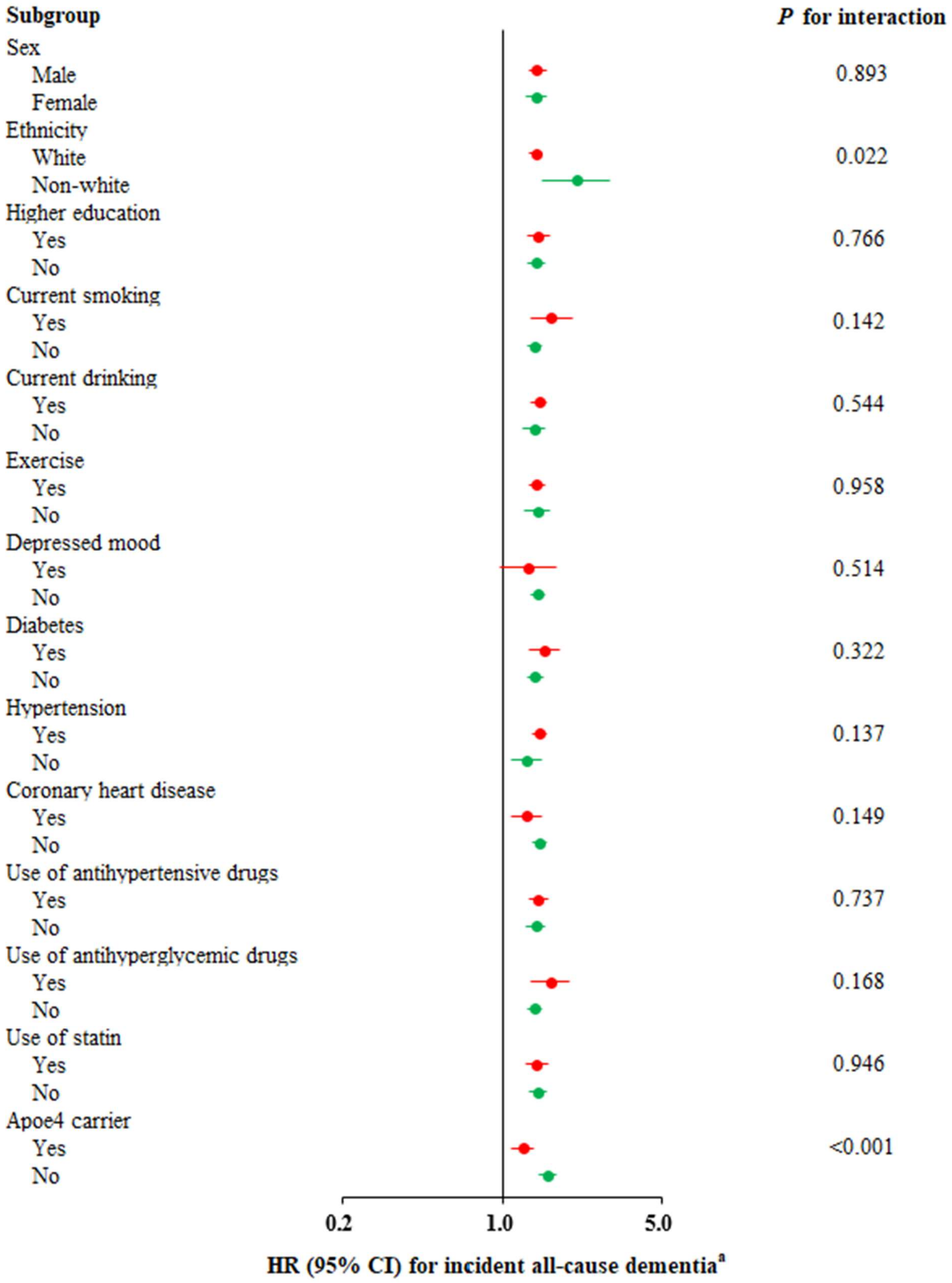

[a]Adjusted for age, sex, race, education, baseline body mass index, low-density lipoprotein cholesterol, current smoking, current drinking, exercise, depressed mood, hypertension, diabetes, coronary heart disease, use of antihypertensive drugs, use of antihyperglycemic drugs, use of statin, and ApoE4 status, except where an

© 2023 Zhang W et al. *JAMA Network Open.*

adjusting variable itself was being tested, by using Cox proportional hazards models.

eFigure 2. Subgroup Analyses to Identify Potential Modifying Effects From Covariates on the Association Between Age at Diagnosis of Atrial Fibrillation (per 10 Years Decrease) and Incident All-Cause Dementia by Using Cox Proportional Hazards Models (n=30 601)

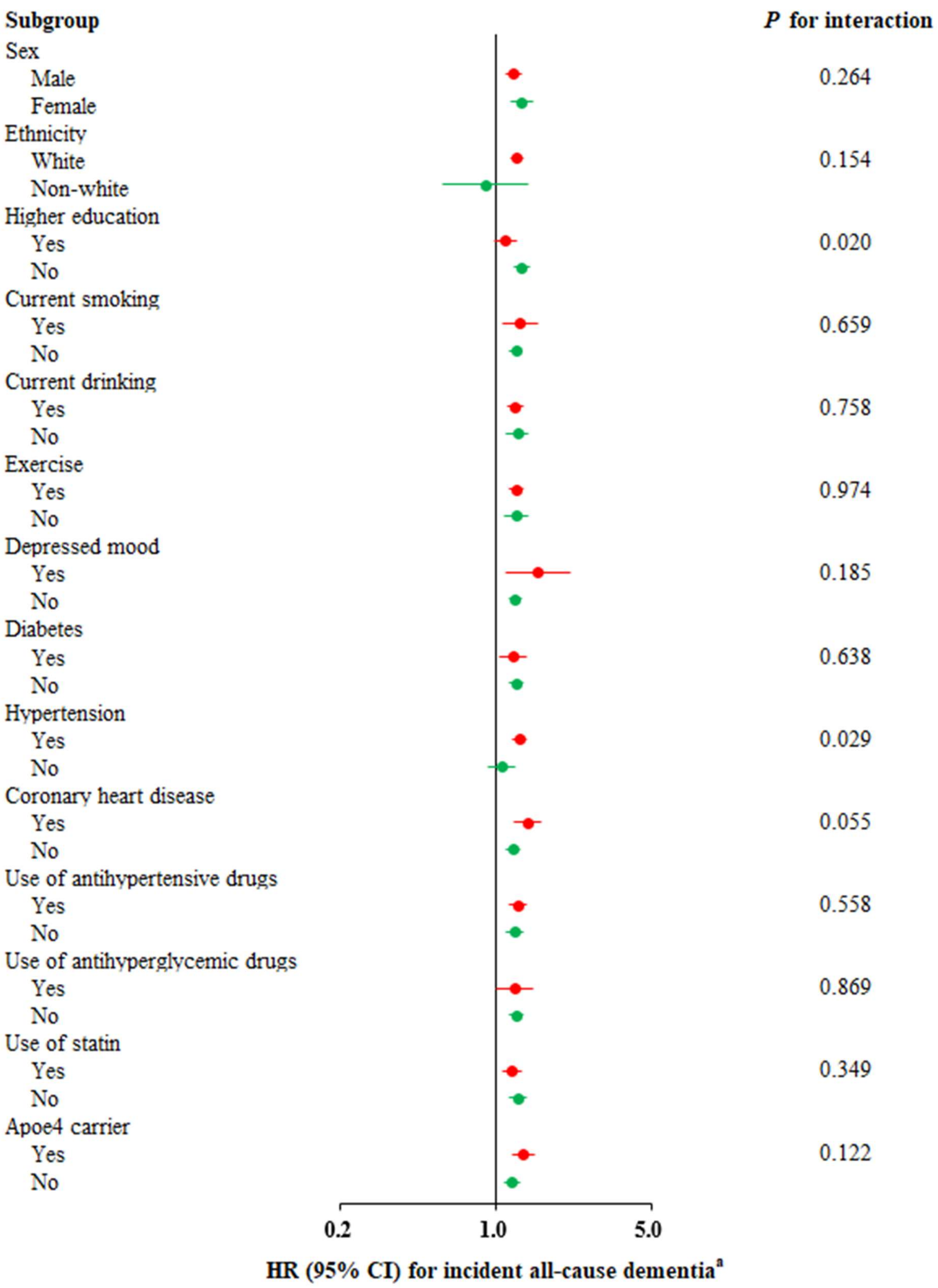

[a]Adjusted for age, sex, race, education, baseline body mass index, low-density lipoprotein cholesterol, current smoking, current drinking, exercise, depressed mood, hypertension, diabetes, coronary heart disease, use of

© 2023 Zhang W et al. *JAMA Network Open.*

antihypertensive drugs, use of antihyperglycemic drugs, use of statin, and ApoE4 status, except where an adjusting variable itself was being tested, by using Cox proportional hazards models

eFigure 3. Subgroup Analyses to Identify Potential Modifying Effects From Covariates on the Association Between Atrial Fibrillation (AF) And Incident All-Cause Dementia in Participants With AF Diagnosed at Younger Than 65 Years and Their Controls by Using Cox Proportional Hazards Models (n=32 061)

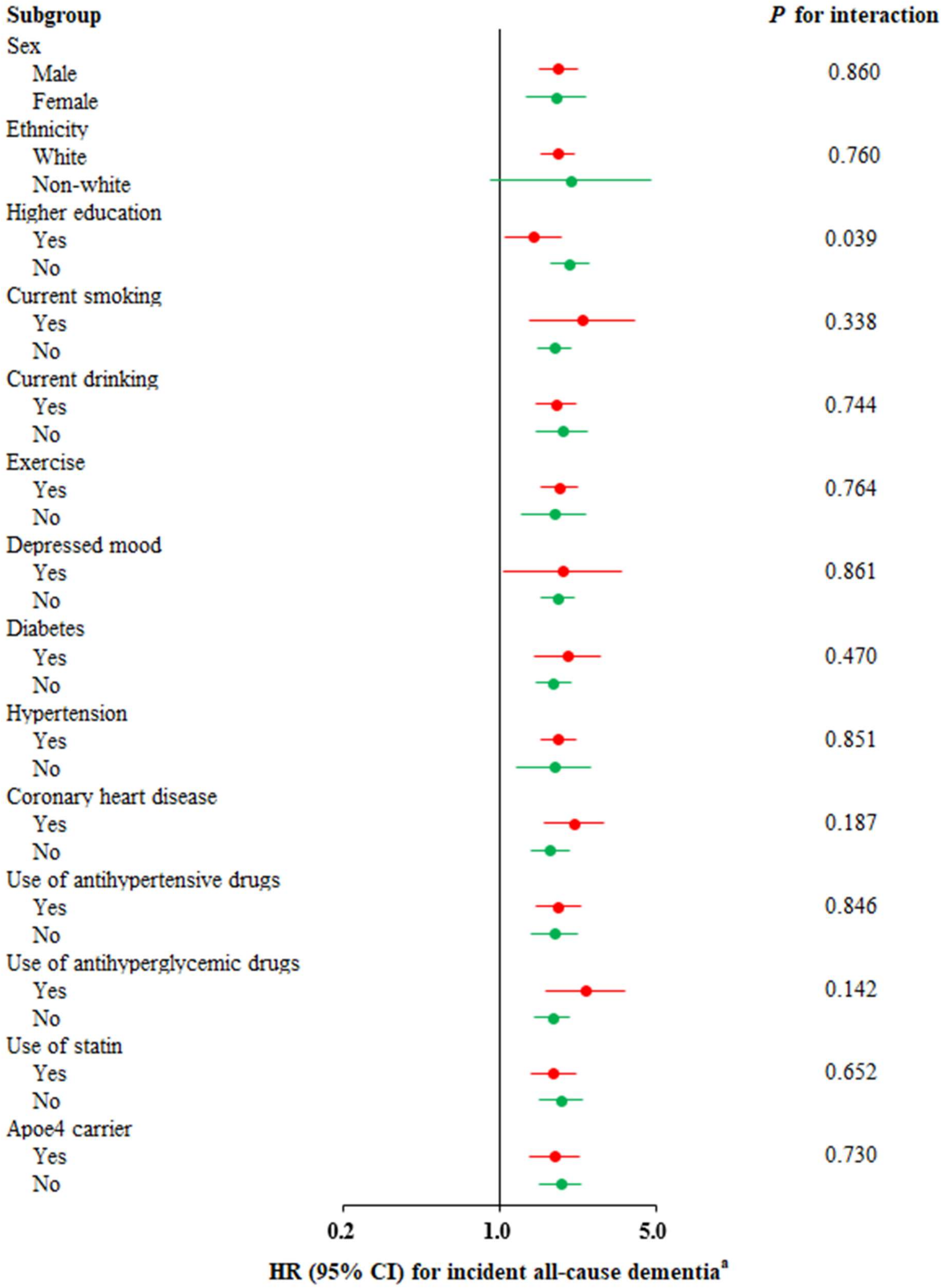

[a]Adjusted for age, sex, race, education, baseline body mass index, low-density lipoprotein cholesterol, current

© 2023 Zhang W et al. *JAMA Network Open.*

smoking, current drinking, exercise, depressed mood, hypertension, diabetes, coronary heart disease, use of antihypertensive drugs, use of antihyperglycemic drugs, use of statin, and ApoE4 status, except where an adjusting variable itself was being tested, by using Cox proportional hazards models.

eFigure 4. Subgroup Analyses to Identify Potential Modifying Effects From Covariates on the Association Between Atrial Fibrillation (AF) and Incident All-Cause Dementia in Participants With AF Diagnosed at Age 65 to 74 Years and Their Controls by Using Cox Proportional Hazards Models (n=41 790)

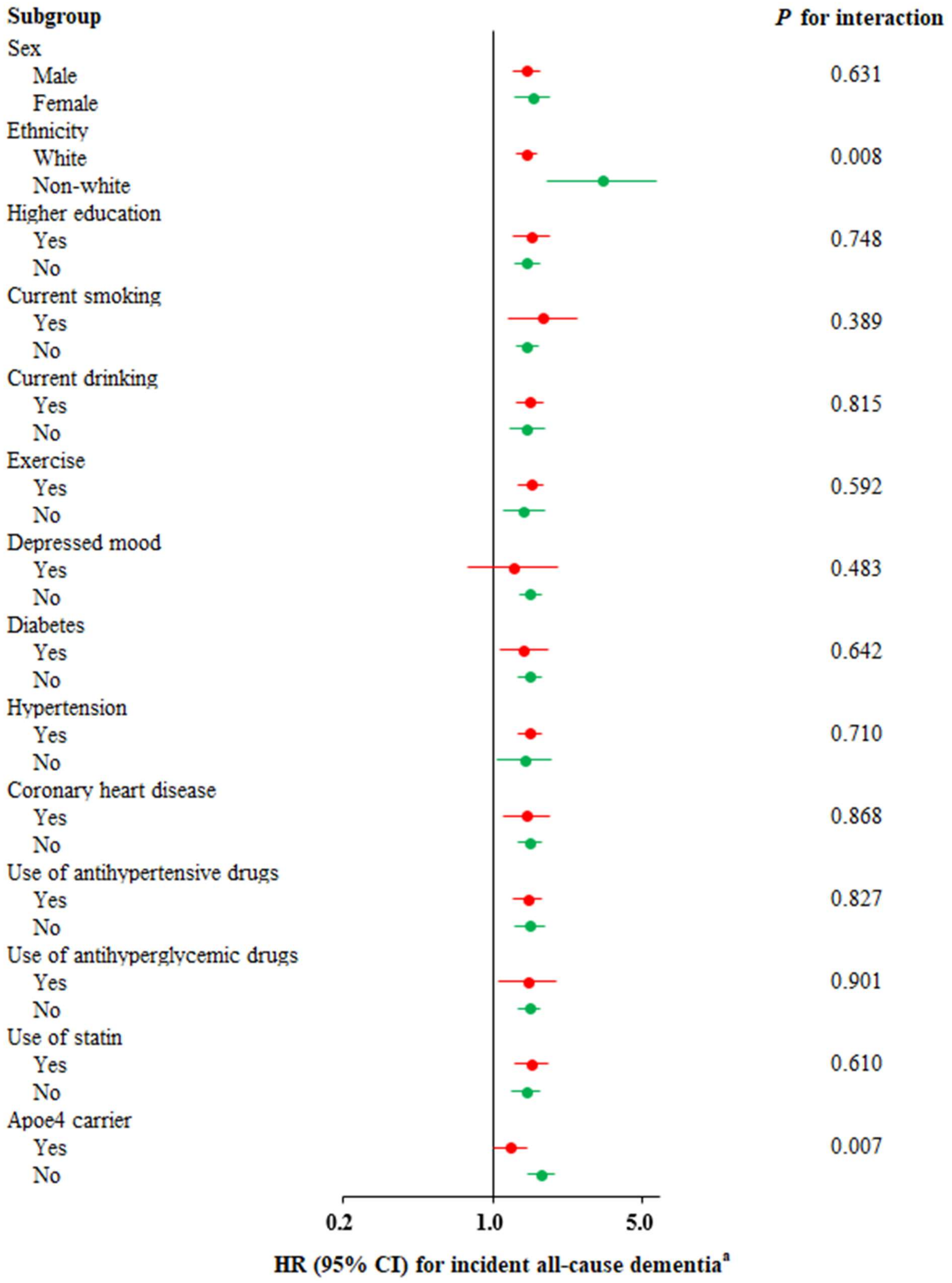

© 2023 Zhang W et al. *JAMA Network Open.*

[a]Adjusted for age, sex, race, education, baseline body mass index, low-density lipoprotein cholesterol, current smoking, current drinking, exercise, depressed mood, hypertension, diabetes, coronary heart disease, use of antihypertensive drugs, use of antihyperglycemic drugs, use of statin, and ApoE4 status, except where an adjusting variable itself was being tested, by using Cox proportional hazards models.

eFigure 5. Subgroup Analyses to Identify Potential Modifying Effects From Covariates on the Association Between Atrial Fibrillation (AF) and Incident All-Cause Dementia in Participants With AF Diagnosed at Age 75 Years and Older and Their Controls by Using Cox Proportional Hazards Models (n=17 949)

© 2023 Zhang W et al. *JAMA Network Open.*

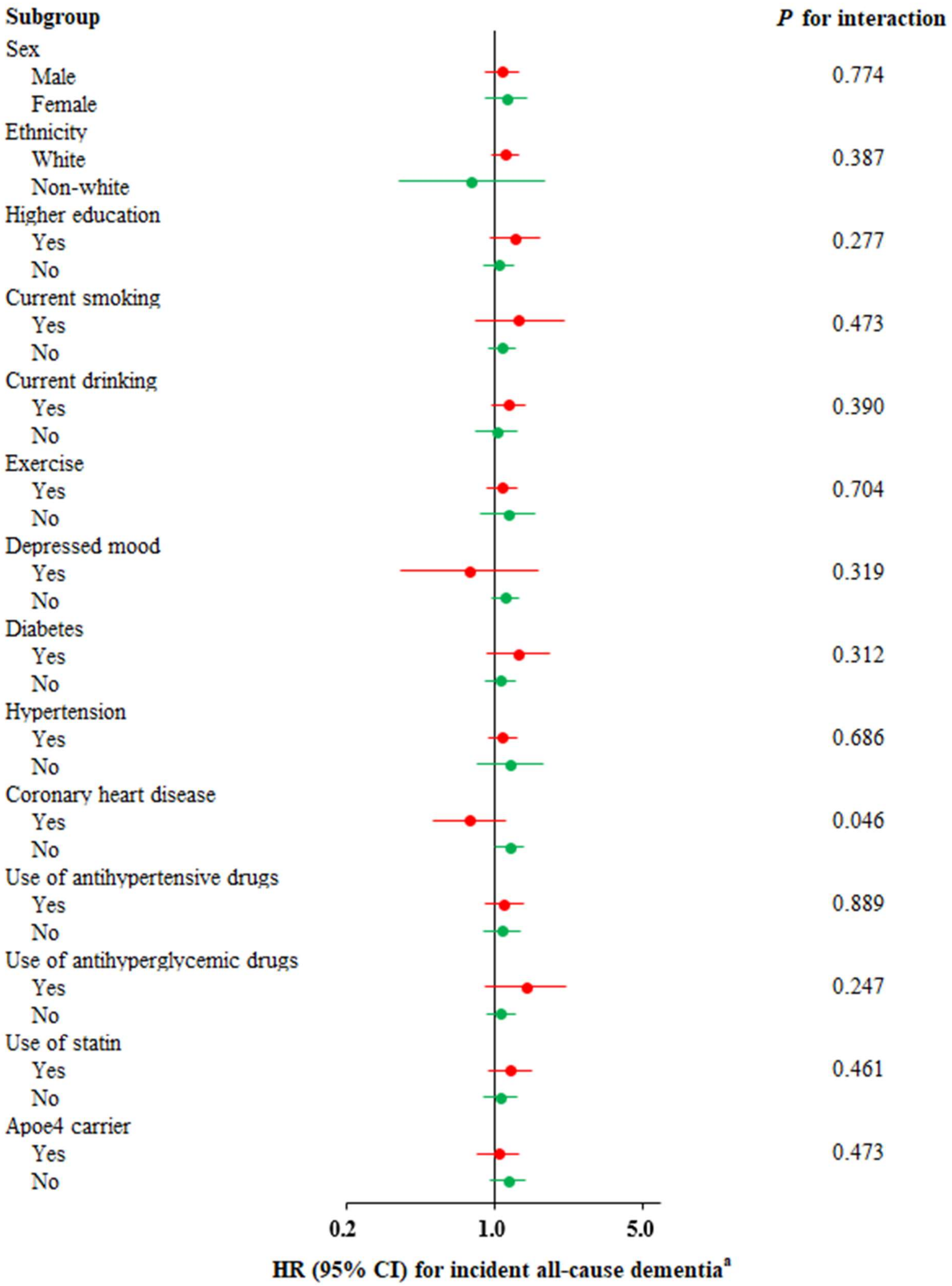

[a]Adjusted for age, sex, race, education, baseline body mass index, low-density lipoprotein cholesterol, current smoking, current drinking, exercise, depressed mood, hypertension, diabetes, coronary heart disease, use of antihypertensive drugs, use of antihyperglycemic drugs, use of statin, and ApoE4 status, except where an adjusting variable itself was being tested, by using Cox proportional hazards models.

© 2023 Zhang W et al. *JAMA Network Open.*
