# Supplementary material for: Age at Diagnosis of Atrial Fibrillation and Incident Dementia
Source: JAMA Netw Open. 2023 Nov 8;6(11):e2342744. doi: 10.1001/jamanetworkopen.2023.42744 (PMC10632957; doi:10.1001/jamanetworkopen.2023.42744)
Supplement: Supplement 2. — Data Sharing Statement [file jamanetwopen-e2342744-s002.pdf]

Data Sharing Statement

Zhang. Age at Diagnosis of Atrial Fibrillation and Incident Dementia. *JAMA Netw Open*.
Published November 10, 2023. doi:10.1001/jamanetworkopen.2023.42744

Data
Data available: No
